# Blocking mechanosensitive ion channels eliminates the effects of applied mechanical loading on chick joint morphogenesis

**DOI:** 10.1098/rstb.2017.0317

**Published:** 2018-09-24

**Authors:** Cristian Parisi, Vikesh V. Chandaria, Niamh C. Nowlan

**Affiliations:** Department of Bioengineering, Faculty of Engineering, Imperial College London, South Kensington Campus, London SW7 2AZ, UK

**Keywords:** joint development, developmental biomechanics, mechanotransduction, calcium ion channels, bioreactor

## Abstract

Abnormalities in joint shape are increasingly considered a critical risk factor for developing osteoarthritis in life. It has been shown that mechanical forces during prenatal development, particularly those due to fetal movements, play a fundamental role in joint morphogenesis. However, how mechanical stimuli are sensed or transduced in developing joint tissues is unclear. Stretch-activated and voltage-gated calcium ion channels have been shown to be involved in the mechanoregulation of chondrocytes *in vitro*. In this study, we analyse, for the first time, how blocking these ion channels influences the effects of mechanical loading on chick joint morphogenesis. Using *in vitro* culture of embryonic chick hindlimb explants in a mechanostimulation bioreactor, we block stretch-activated and voltage-gated ion channels using, respectively, gadolinium chloride and nifedipine. We find that the administration of high doses of either drug largely removed the effects of mechanical stimulation on growth and shape development *in vitro*, while neither drug had any effect in static cultures. This study demonstrates that, during joint morphogenesis, mechanical cues are transduced—at least in part—through mechanosensitive calcium ion channels, advancing our understanding of cartilage development and mechanotransduction.

This article is part of the Theo Murphy meeting issue ‘Mechanics of development’.

## 1. Introduction

Developmental joint disorders, such as developmental dysplasia of the hip, arthrogryposis and femoroacetabular impingement, constitute abnormal formation of the shape of the joint as it grows (reviewed in [1,2]), and joint shape is increasingly thought to be a critical factor in the risk of osteoarthritis in later life [3]. It has been shown that joint morphogenesis is significantly influenced by mechanical forces, particularly those due to fetal movements (reviewed in [4]). Altered mechanical stimulation due to a lack of, or abnormal, fetal movements in animal models leads to abnormal joint shape development, among other effects including delayed ossification and congenital scoliosis [5–9]. Using an *in vitro* limb explant culture system, we previously showed that a loading regime replicating flexion movements observed *in ovo* can promote features of normal joint morphogenesis [10].

The underlying mechanisms and factors regulating the transduction of mechanical cues in biological responses during joint shape development are still being explored. Brunt *et al*. have shown that, in the zebrafish jaw joint, canonical Wnt signalling pathways (particularly Wnt16) are involved in the transduction of mechanical signals affecting proliferation and migration, important for the shaping of the jaw joint [11]. A recent study (using chick and mouse models) also described a mechanoregulatory role for canonical Wnt signalling during joint development, linked to downregulation of BMP signalling [12]. At the level of the cell membrane, mechanosensitive ion channels are likely to play a fundamental role in mechanotransduction and regulation of cartilage development. Changes in intracellular sodium, potassium and, especially, calcium ion concentrations are considered one of the first signalling events in response to mechanical stimulation of chondrocytes [13,14]. When chondrocytes are mechanically deformed *in vitro*, a flux of ions is generated across the cells membranes, and growth and differentiation of cells are significantly influenced (reviewed in [15]). Cell membrane ion channels are gated passageways for ions that cross the cell membrane and regulate intracellular and extracellular ion concentrations responding to external mechanical stimuli [16] and are thought to be involved in cell volume regulation and cell division [17]. Stretch-activated and L-type (long-lasting activation) voltage-gated calcium ion channels have been shown to be involved in the regulation of cell volume in isolated chondrocytes following induced deformations (reviewed in [15]). However, the role of mechanosensitive ion channels in mechanotransduction in the growth or morphogenesis of developing cartilage has not yet been investigated.

In this study, we investigate the role of two calcium ion channels with mechanosensitive activities in the mechanoregulation of joint morphogenesis in the embryonic chick. Using our previously described *in vitro* mechanostimulation bioreactor system, we use blocking agents, gadolinium chloride and nifedipine, to inhibit, respectively, cation-selective stretch-activated or L-type calcium ion channels, and investigate the effects of blocking the channels on joint shape under static and dynamic loading conditions. It was hypothesized that the mechanical stimuli direct localized joint shape development and are transduced in part through the function of mechanosensitive stretch-activated and voltage-gated calcium ion channels.

## 2. Methods

### (a) Limbs explant preparation and culture

Fertilized white DeKalb eggs (Henry Stewart & Co. Ltd, UK) were incubated at 37°C under humidified conditions. After 7 days of incubation, the chick embryos were harvested, their hindlimbs dissected, and the surrounding soft tissues removed in order to improve the culture efficiency and limit necrosis as described previously [10]. Limb explants were placed on rectangular polyurethane foam supports (Sydney Heath & Son, UK) and cultured in a mechanostimulation bioreactor (Ebers TC-3, Spain) for 6 days [10]. Alpha-minimum essential medium (α-MEM GlutaMAX, Thermo Fisher Scientific, USA) supplemented with 1% penicillin/streptomycin/amphotericin B and 100 µM ascorbic acid (Sigma Aldrich, USA) was used for the culture. The function of cation-selective stretch-activated ion channels was blocked using gadolinium chloride (GdCl_3_) dissolved in 1 M hydrochloric acid (HCl) (Sigma Aldrich, USA). Voltage-gated calcium ion channels were blocked using nifedipine (Nf) dissolved in DMSO (Sigma Aldrich, USA). Blocking agents were added to the complete basal media at two concentrations based on previously used doses in cell cultures: a low dose of 0.1 mM and a high dose of 1 mM for both ion channel blockers [18]. Mouw *et al*. tested GdCl_3_ or Nf dosages at concentrations varying from 0.001 to 10 mM in three-dimensional agarose gels seeded with isolated chondrocytes [18]. Based on their results, we considered the concentrations of 0.1 and 1 mM to be the most appropriate for tissue culture and these concentrations were therefore used in this study. The final concentration of the GdCl_3_ vehicle (HCl) was 0.5 mM, and the final concentration of the Nf vehicle was 0.7 mM. Vehicle control group experiments (without any drug) were also performed at the same concentrations of HCl and DMSO to assess the impact of the solvents used. The explants were maintained at an air–liquid interface, and the culture medium was replenished every 24 h.

### (b) Mechanical stimulation

**Figure 1.**
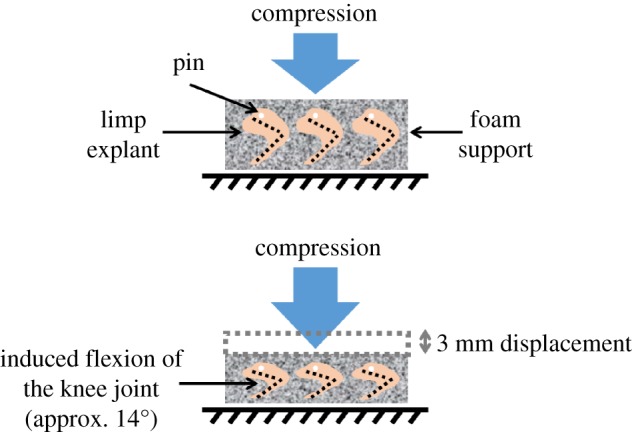
Schematic of the mechanical loading system in the bioreactor culture chamber. A cyclic 3 mm compressive displacement was applied to the foam, which induced a flexion of the limbs of approximately 14°.

As employed previously, the bioreactor was programmed to apply a 3 mm compressive displacement regime to the foam supports in order to induce an approximate 14° change in angle between the thigh and shank of the limbs (figure 1), mimicking a normal/physiological *in ovo* flexion motion of the knee joint of the chick embryo [10]. The dynamic compression was applied to all explants for 2 h, three times per day, in a sinusoidal waveform at a frequency of 0.67 Hz. Static cultures were performed using the same set-up but without any mechanical stimulation to confirm the interaction between ion channels and mechanical stimulation.

### (c) Analysis

Three experimental groups for each blocking agent (low dose, high dose and vehicle control) were obtained, for both dynamic and static cultures, leading to 12 groups in total, as described in table 1. Eight or nine knee (stifle) joints were cultured and analysed for each group (table 1), obtained from two separate cultures which were pooled. During media changes, each culture chamber was visually inspected for any sign of abnormal growth or infection, none of which was observed in these experiments. After 6 days, the cartilaginous skeletal rudiments of each explant were stained with 0.055% Alcian Blue solution for 5 h and cleared in 1% KOH for approximately 15 min at room temperature, as described previously [10].

**Table 1.** Number of specimens analysed for each experimental group.

	dynamic cultures	static cultures
	number of specimens	
GdCl_3_ vehicle ctrl (0.5 mM HCl)	9	9
GdCl_3_ low dose (0.1 mM)	9	9
GdCl_3_ high dose (1 mM)	9	9
Nf vehicle ctrl (0.7 mM DMSO)	9	9
Nf low dose (0.1 mM)	8	9
Nf high dose (1 mM)	9	9

Samples were imaged in three dimensions using Optical Projection Tomography (OPT) [19] following protocols described by Quintana and Sharpe [20]. The sample was reconstructed using NRecon software (Bruker, USA) and the three-dimensional models of the limbs were rendered using Mimics Research 19.0 software (Materialise, Belgium). Frontal, lateral and medial views of the joint were traced to produce shape profiles for each specimen. Detailed measurements of width, height and depth of the distal femur, proximal tibia and fibula were performed using ParaView v. 5.2.0 software (Kitware Inc., USA) as described previously [10].

One-way analysis of variance (ANOVA) tests were performed to verify if significant differences existed within the groups. When significant differences were found, a *post-hoc* Tukey pairwise comparison was performed to identify which groups were significantly different from each other. When the normality test failed, a non-parametric *post-hoc* Kruskal–Wallis test was performed. *P*-values less than 0.05 were considered statistically significant. For all measurements in which there was at least one significant difference between groups, the *p*-values are provided in the electronic supplementary material, Data, tables S1–S10.

## 3. Results

### (a) Effect of cation-selective stretch-activated ion channels

**Figure 2.**
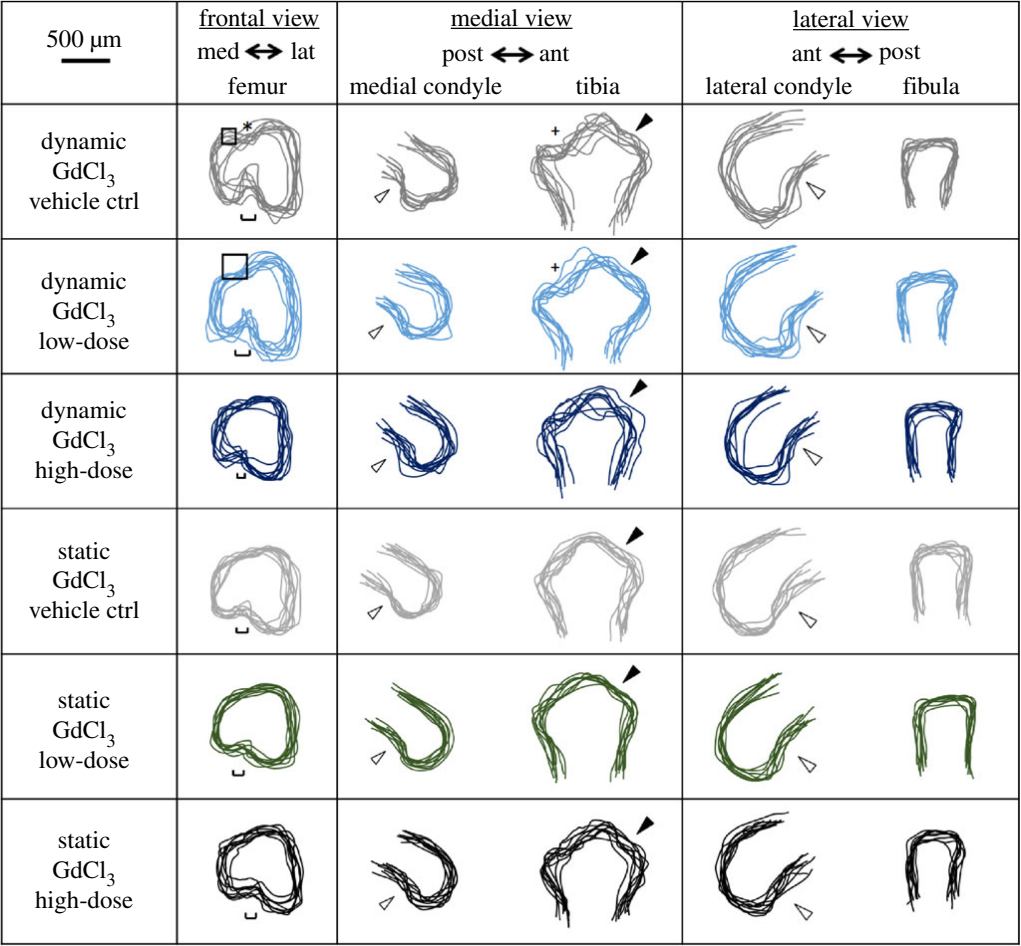
Blocking of stretch-activated ion channels using a high dose of gadolinium chloride (GdCl_3_) during dynamic culture had noticeable effects on joint shapes compared to dynamic vehicle controls, with the knee joints of the dynamic high-dose group being more similar to the static groups. Administration of the drug during static cultures had no obvious effects on shape. Low dose, 0.1 mM; high dose, 1 mM; squares, dominance of the femoral lateral condyle over the medial condyle; hollow arrows, posterior curl of condyles seen in moved explants; *, dorsal groove between the two femoral condyles; single-edged bars, intercondylar fossa width; +, concave curve of the tibial plateau; filled arrows, anterior tibial crest.

**Figure 3.**
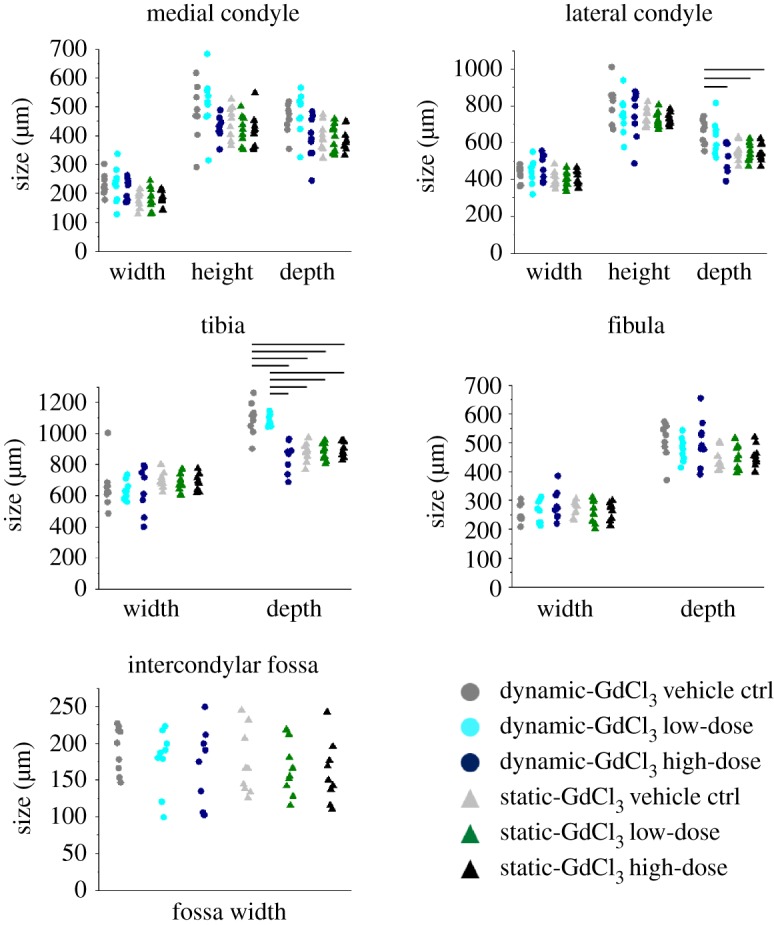
Blocking of stretch-activated ion channels using a high dose of gadolinium chloride (GdCl_3_) during dynamic cultures led to a significant reduction in tibial depth and lateral condyle depth. No measurements were significantly different between the dynamic high-dose group and any of the static groups. Circle (for dynamic cultures) and triangles (for static cultures) represent individual data points. Black horizontal lines represent significant differences between groups (significance for *p*-value less than 0.05). Full details of statistical results, including means, standard deviations (s.d.) and *p*-values, are reported in the electronic supplementary material, tables S1–S10.

The gadolinium chloride vehicle control (0.5 mM HCl) did not have substantial effects on the previously described effects of dynamic stimulation on joint shape development *in vitro* [10]. In brief, previously described features of joint shapes cultured under dynamic stimulation [10] were seen in the dynamic vehicle control group, including a clear intercondylar fossa in the frontal view of the distal femur, a dorsal groove between the condyles on the superior surface of the rudiment, posterior curls in both the medial and lateral condyles, and development of the anterior tibial crest and concave tibial plateau (figure 2). In explants cultured under static conditions, there was no clear separation between medial and lateral condyle in the frontal view, with a noticeable underdevelopment of the medial condyle compared to dynamic vehicle controls, and a reduction in the definition of the tibial plateau profile in the medial view (figure 2). However, the only significant difference between the dynamic and static vehicle control groups was a reduction in the tibial depth in the static group (figure 3), compared to the five significant measurements between static and dynamic shapes in our previous study. This may indicate that the vehicle (HCl) had a general effect on growth. Based on the commonality in changes in shape features between static and dynamic cultures as described above (figure 2) and previously [10], and the trends towards decreased measurements in the static vehicle group (figure 3), we were confident that the vehicle was not substantially affecting the response of the tissues to mechanical stimulation *in vitro*.

When a low dose (0.1 mM) of GdCl_3_ was added to the media during dynamic cultures, the developing explants appeared only slightly altered in their resultant joint shapes compared to dynamic vehicle controls. In the frontal shape profile of the distal femur, an increased offset between the top of the femoral condyles was noticed along with the absence of the dorsal femoral groove (figure 2). The femoral condyles, the anterior tibial crest and tibial plateau all appeared similar to dynamic vehicle controls, showing no difference in the medial and lateral shape profiles of the joint (figure 2). Measurements of explants cultured with the low dose of GdCl_3_ did not show any significant difference to vehicle controls (figure 3).

Adding the high dose (1 mM) of GdCl_3_ to the culture media led to noticeable effects on joint shape compared with dynamic vehicle control and low-dose groups, with a similar underdevelopment as was seen under static culture. In the frontal view, both femoral condyles appeared merged together with no clear sign of separation at the intercondylar fossae, which were significantly underdeveloped. The posterior curls of medial and lateral condyles were similar to dynamic vehicle controls and low-dose groups. The shapes of the proximal tibiae were also noticeably different, with no clear sign of the anterior tibial crests or tibial plateaus (figure 2). Tibial depth was significantly less in the high-dose dynamic group compared with the dynamic vehicle control and low-dose groups, while lateral condyle depth was decreased in the high-dose group compared with dynamic vehicle controls (figure 3). The joint shapes that resulted from the high dose of the drug under dynamic stimulation were, therefore, very similar to shapes and sizes obtained under static conditions either with vehicle, low-dose or high-dose drug. Indeed, no significant differences in measurements were found between the dynamic GdCl_3_ high-dose group and any static group. Therefore, (i) GdCl_3_ at high dose had no effect on joint shape when limbs were cultured in static conditions and (ii) the drug at high dose effectively removed the effects of dynamic stimulation on joint shape.

### (b) Effect of L-type voltage-gated calcium ion channels

**Figure 4.**
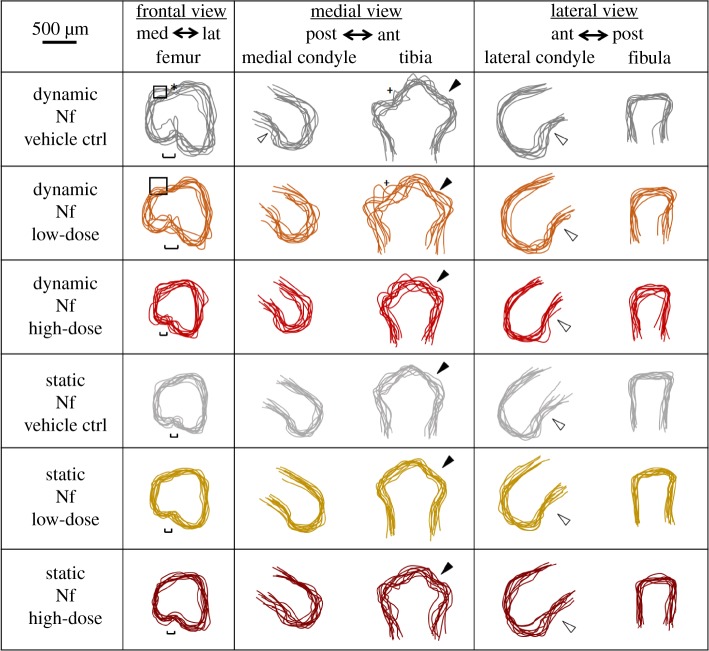
Blocking of voltage-gated ion channels using a high dose of nifedipine (Nf) during dynamic culture had noticeable effects on joint shapes compared with dynamic vehicle controls, with the knee joints of the high-dose group being more similar to the static groups. Administration of the drug during static cultures had no obvious effects on shape. Low dose, 0.1 mM; high dose, 1 mM; squares, dominance of the lateral condyle over the medial condyle; hollow arrows, posterior curl of condyles seen in moved explants; *, dorsal groove between the two femoral condyles; single-edged bars, intercondylar fossa width; +, concave curve of the tibial plateau; filled arrows, anterior tibial crest.

**Figure 5.**
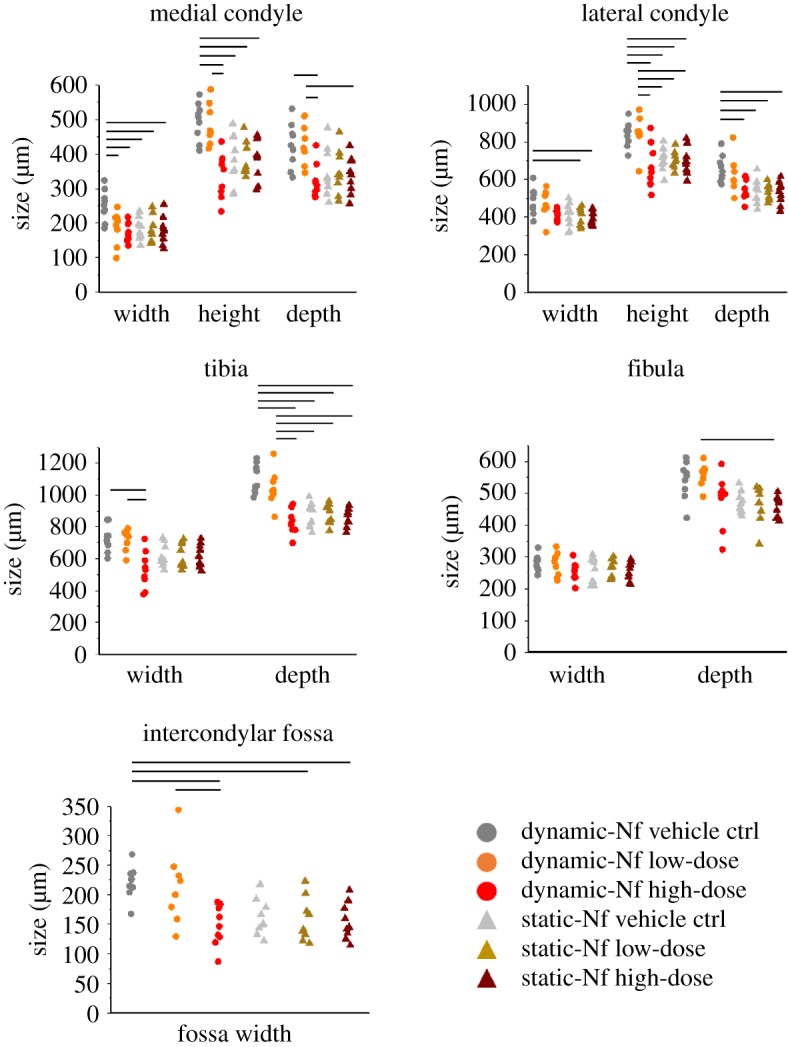
Blocking of voltage-gated ion channels using a high dose of nifedipine (Nf) during dynamic cultures led to significant reductions in 8 out of 11 measurements performed. No measurements were significantly different between the dynamic high-dose group and any of the static groups. Circle (for dynamic cultures) and triangles (for static cultures) represent individual data points. Black horizontal lines represent significant differences between groups (significance for *p*-value less than 0.05). Full details of statistical results, including means, standard deviations (s.d.) and *p*-values, are reported in the electronic supplementary material, tables S1–S10.

Similar to what was found for the gadolinium chloride vehicle, DMSO (0.7 mM) as the vehicle for nifedipine did not substantially alter the previously described effects on the joint shape of dynamic stimulation compared to static cultures [10]. After dynamic culture, separation of the femoral condyles was clear and the shape of the intercondylar fossa was well defined. The dorsal groove on the superior side, the posterior curl of the femoral condyles, the formation of the anterior tibial crest, tibial plateau and flattened profile of the fibula appeared normally developed (figure 4). In explants cultured under static conditions with the vehicle, no clear separation between condyles was seen, with a significant underdevelopment of the medial condyle. A reduction in the definition of the posterior curl of the medial condyle and of the tibial plateau profile was found in the medial view of the joint (figure 4). Significant reductions of the average widths, heights and depths of medial and lateral condyles, depth of tibiae and fibulae and widths of intercondylar fossae confirmed the visible shape differences previously found (figure 5). Therefore, there were more quantitative differences between the static and dynamic joint shapes with the nifedipine vehicle than in our previous study [10], indicating that DMSO may—like HCl—have effects on growth in general. However, as previously, overall changes in shape features due to dynamic culture were maintained despite the vehicle.

Separation of the femoral condyles appeared to be only subtly affected following the administration of the low dose (0.1 mM) of nifedipine during the dynamic cultures, with both the femoral intercondylar fossa and dorsal groove being slightly more difficult to distinguish in the frontal profile compared with the dynamic vehicle control group (figure 4). The medial condyle also appeared to be partially underdeveloped by blocking the calcium ion channels, with no posterior curl evident and a flattened articular end compared to controls (figure 4). The tibial plateau was not distinguishable in the medial view for some explants (figure 4). However, no statistically significant differences in joint region size were found following the low-dose administration of nifedipine compared with dynamic vehicle controls (figure 5).

Similarly to the blocking of cation-selective stretch-activated ion channels, the administration of a high dose (1 mM) of nifedipine during the dynamic culture considerably affected joint shape development, with similar shape features seen in static cultures. The distal femur appeared simplified with no clear definition of each individual condyle in the frontal view (figure 4). The medial condyle appeared significantly smaller compared with vehicle controls and the Nf low-dose group (figure 4). The posterior curl of the medial condyle was less pronounced in the medial view, and the concavity of the tibial plateau profile was less defined (figure 4). However, the formation of the anterior tibial crest did not appear to be affected, and the lateral view of the joint did not reveal any major differences in joint shape. Most measured joint regions were significantly decreased following a high dose of calcium ion channel blocker in dynamic cultures compared with the dynamic vehicle control (medial condyle width, height and depth; lateral condyle height and depth; tibia width and depth; intercondylar fossa width) and dynamic low-dose (medial condyle height and depth; lateral condyle height; tibia width and depth; intercondylar fossa width) cultures (figure 5). There were no significant differences between any of the measurements of the dynamic high dose and those of any of the static groups (figure 5). Therefore, similar to the findings for gadolinium chloride, (i) nifedipine at high dose had no effect on joint shape when limbs were cultured in static conditions and (ii) the drug at high dose effectively removed the effects of dynamic stimulation on joint shape.

### (c) Comparative effects of blocking cation-selective stretch-activated versus L-type voltage-gated calcium ion channels

As described above, both drugs at high dosage had significant effects on the development of the knee joint in dynamic cultures. The cultures in which the voltage-gated calcium ion channels were blocked (using nifedipine) exhibited more statistically significant differences (compared to dynamic vehicle controls) in joint shape measurements than was found for the gadolinium chloride cultures, in which stretch-activated ion channels were blocked. However, there were no significant differences between the two high-dose dynamic groups (electronic supplementary material, tables S1–S10).

## 4. Discussion

In this study, the functions of cation-selective stretch-activated or L-type voltage-gated calcium ion channels were inhibited by, respectively, gadolinium chloride and nifedipine during dynamic and static cultures of explanted chick embryo hindlimbs. In dynamic cultures, the shape of the knee joint following administration of high doses of either blocking agent was substantially altered compared with vehicle controls. Joint shapes that developed under dynamic culture in which the ion channels were blocked were similar to those which developed under static culture, whether with or without high-dose blocking agents. As described previously, the joint shapes that grow and form in our *in vitro* culture system are not equivalent to normally developed joints [10]. However, key shape features seen in normally developing joints emerge when limbs are cultured under mechanical loading, such as clear separation of the condyles, the formation of tibial plateau and crest and definition of the posterior curls of medial and lateral condyle [10]. These features of normal shape development are lost when high doses of either ion-channel-blocking drug were applied. These findings suggest that the inactivity of the two selected ion channels largely removed the effects of dynamic stimulation in developing explants, while not showing any appreciable effect in static culture conditions. This, therefore, provides evidence for the involvement of both stretch-activated and L-type calcium ion channels in mechanotransduction of joint morphogenesis.

We are not aware of previous studies in which mechanosensitive ion channels were blocked in whole tissue explant cultures. However, the results presented here are in agreement with studies of dynamic cultures of isolated chondrocytes in which mechanosensitive ion channels were blocked using GdCl_3_ or Nf [18,21–25]. The presence of gadolinium inhibited the increase of intracellular Ca^++^ concentration due to the application of mechanical stimuli [21,22], suggesting the Ca^++^ influx through stretch-activated calcium ion channels was disrupted by the presence of the drug. This would also mean that the consequent release of intracellular messengers and the activation of kinase cascades due to the Ca^++^ influx would be affected [18]. Nifedipine used in chondrocyte cultures has also been reported to inhibit calcium influx, influencing cellular mechanisms regulated by the Ca^++^ oscillations that are involved in cell proliferation and differentiation [18,23,24,26]. Therefore, the reduction of joint growth seen in the distal femur and proximal tibia in this study when either of the selected drugs were applied could be due to a decrease of calcium ion influx and the consequent inhibition of those related mechanisms. Mechanically induced proliferation, differentiation and extracellular matrix synthesis have also been reported to decrease in the presence of gadolinium or nifedipine, representing plausible scenarios for the altered development of the knee joint found in this study [23–26].

The drug dosages used in this research (0.1 mM for ‘low dose’ and 1 mM for ‘high dose’) were selected on the basis of a previous study of three-dimensional cultures of isolated chondrocytes [18]. Mouw *et al*. [18] showed that the presence of GdCl_3_ or Nf significantly affects mechanotransduction in bovine chondrocytes seeded in three-dimensional agarose gels. The authors tested drug dosages at concentrations varying from 0.001 to 10 mM, and found the most significant reduction of protein synthesis in dynamic cultures with doses of 0.1 and 1 mM for either drug [18]. These doses were higher than in other studies of two-dimensional cell monolayers, in which drugs were administrated at the concentrations of 0.01 or 0.02 mM [21–25], but GdCl_3_ was used at a concentration of 0.1 mM by Kerrigan and Hall in a study with bovine chondrocytes cultured in two dimensions as a cell monolayer [27] and Nf at the same concentration of 0.1 mM with chicken chondrocytes by Zuscik *et al*. [28]. Considering the differences in cell number and density between whole-limb and cell monolayer cultures, we selected the most significant concentrations of the drugs (0.1 and 1 mM) as found by Mouw *et al*. [18] in their three-dimensional cultures. This selection has been confirmed as appropriate by our results because the 0.1 mM dose did not lead to any significant effects as compared to vehicle controls (for either drug) and the higher dose of 1 mM (for either drug) showed an effect only when the mechanical stimulation was applied, with no effects of any sort in static cultures.

There are some limitations to this study. Since the performance of the ion channel blockers was not assessed during these experiments, it is not known whether the ion channels were completely or partially blocked. However, both blocking agents have repeatedly been used to block the activity of the selected ion channels in cultures of isolated chondrocytes [18,21–24], and we assume that they operate in a similar way in tissue explant cultures. In future studies, we aim to assess the activation of the ion channels by the mechanical loading in the explanted limb tissue, using (for example) fluorescence-based methods, such as an ion flux assay for stretch-activated channels, or a voltage-sensitive assay for voltage-gated channels [29]. There are no clear studies on off-target effects on chondrocytes. Off-target effects of the drugs are discussed in the literature, and include the induction of apoptosis or necrosis of fibroblasts or neural cells for GdCl_3_, and the toxicity of Nf for neural or liver cells and its effect on the cardiovascular system [23,25,30–32]. While proliferation and differentiation of chondrocytes have been shown to be affected by GdCl_3_ or Nf, this is usually attributed to the main mechanism of action of the drugs on Ca^++^ influx [18,23–26]. Substantial off-target effects due to each drug in our study seems unlikely, due to the absence of any effect in static cultures even when the high (1 mM) dose was used. We can therefore speculate that off-target effects, if present, have not played any significant direct role in affecting the joint morphogenesis in our *in vitro* model, otherwise we should have found some effects in the static cultures.

A slightly more severe underdevelopment was observed following the administration of nifedipine at high dose compared with gadolinium chloride, with more significant differences among groups within the same drug but no significant differences between the two drugs (electronic supplementary material, tables S1–S10). This may be due to the different mechanisms of action of gadolinium and nifedipine. Gadolinium chloride influences the duration of ion channel opening, while nifedipine maintains inactivity of closed channels [33–35], hence the inhibition of the ion influx with nifedipine may be more effective. It is also possible that the vehicles used for each of the drugs could have had some effects on growth, as subtle differences were seen in the size of the effect on shape due to dynamic culture in vehicle control cultures, compared to our previous study in basal media [10]. However, while the number of significantly different measurements (between static and dynamic) varied between the basal media and the two vehicle control groups, the key shape features resulting from dynamic stimulation were consistent between these three groups. Future studies on the distribution of mechanical strain could enhance the understanding of the differential responses to loading in the different anatomical locations. Some preliminary work has been done in the group towards developing computational models to analyse the distribution of stress and strain in the rudiments when mechanically stimulated in our bioreactor system, but further work is needed, which will inform our understanding of this work. Other future studies may involve the use of other drugs that specifically target stretch-activated or voltage-gated calcium ion channels, in order to more firmly establish the effects of GdCl_3_ and Nf. These might include spider venom peptide GsMTx4, for stretch-activated ion channels [36,37], and Verapamil or diltiazem, for voltage-gated ion channels [38,39]. Finally, genetic factors involved in the mechanotransduction process during joint morphogenesis, such as Wnt and BMP signalling [11,12], have not been investigated here, but the current study paves the way for future studies focusing on gene-level and further organelle-level mechanisms using the *in vitro* system.

In conclusion, we have demonstrated that stretch-activated and voltage-gated calcium ion channels are involved in the mechanoregulation of joint shape development. Following a dynamic movement regime known to stimulate joint morphogenesis *in vitro*, the distal femur and proximal tibia in the embryonic chick knee joint were abnormally shaped when either candidate ion channels were blocked, appearing similar to statically cultured joints. These results agree with previous findings in chondrocyte cell culture studies demonstrating the role of these ion channels in transducing mechanical signals for cartilage growth, and provide new evidence to support the hypothesis that mechanical cues that direct localized joint shape development are transduced through the function of these channels. Tissue engineers aim to recapitulate *in vitro* the processes that occur during development in order to regenerate damaged or diseased tissues, but there has been a limited success in clinical translation of cartilage tissue engineering strategies to date, leading to increased interest in potential crosstalk between developmental biology and tissue engineering [40]. This study has the potential to feed into tissue engineering by providing a better understanding of the mechanisms involved in cartilage tissue development.

## Supplementary Material

Statistical Analysis

## Data accessibility

The electronic supplementary material file associated with this paper (Tables p-values.docx) provides Tables S1–S10 detailing means and standard deviations for all measurements, and *p*-values for all measurements in which there was at least one significant difference between groups.
