# Supplementary material for: Blocking mechanosensitive ion channels eliminates the effects of applied mechanical loading on chick joint morphogenesis
Source: Philos Trans R Soc Lond B Biol Sci. 2018 Sep 24;373(1759):20170317. doi: 10.1098/rstb.2017.0317 (PMC6158207; doi:10.1098/rstb.2017.0317)
Supplement: Statistical Analysis [file rstb20170317supp1.docx]

**Supplementary Data – Statistical analysis**

Tables below list all the *p*-values for all measurements in which there was at least one significant difference between groups.

**Table S1. List of *p*-values obtained with the Tukey test for significant differences in the measurements of medial condyle width.**

|  | **Mean**  **± SD (μm)** | **Dyn GdCl_3_ Vh Ctrl** | **Dyn GdCl_3_ Low Dose** | **Dyn GdCl_3_ High Dose** | **Static GdCl_3_ Vh Ctrl** | **Static GdCl_3_ Low Dose** | **Static GdCl_3_ High Dose** | **Dyn**  **Nf**  **Vh Ctrl** | **Dyn**  **Nf**  **Low Dose** | **Dyn**  **Nf**  **High Dose** | **Static Nf**  **Vh Ctrl** | **Static Nf**  **Low Dose** | **Static Nf**  **High Dose** |
| --- | --- | --- | --- | --- | --- | --- | --- | --- | --- | --- | --- | --- | --- |
| **Mean**  **± SD (μm)** |  | 228.9  ± 36.7 | 228.3  ± 62.3 | 215.2  ± 37.1 | 181.7  ± 29.9 | 182.2  ± 39.6 | 183.4  ± 27.2 | 251.6  ± 44.8 | 186.2  ± 48.8 | 172.8  ± 27.3 | 181.1  ± 31.8 | 179.9  ± 37.6 | 178.6  ± 40.0 |
| **Dyn GdCl_3_**  **Vh Ctrl** | 228.9  ± 36.7 | - | 1.000 | 1.000 | 0.337 | 0.353 | 0.395 | 0.987 | 0.542 | 0.124 | 0.317 | 0.283 | 0.247 |
| **Dyn GdCl_3_**  **Low Dose** | 228.3  ± 62.3 |  | - | 1.000 | 0.359 | 0.376 | 0.418 | 0.983 | 0.567 | 0.135 | 0.338 | 0.303 | 0.265 |
| **Dyn GdCl_3_**  **High Dose** | 215.2  ± 37.1 |  |  | - | 0.819 | 0.833 | 0.865 | 0.726 | 0.936 | 0.506 | 0.800 | 0.765 | 0.721 |
| **Static GdCl_3_**  **Vh Ctrl** | 181.7  ± 29.9 |  |  |  | - | 1.000 | 1.000 | **0.016** | 1.000 | 1.000 | 1.000 | 1.000 | 1.000 |
| **Static GdCl_3_**  **Low Dose** | 182.2  ± 39.6 |  |  |  |  | - | 1.000 | **0.017** | 1.000 | 1.000 | 1.000 | 1.000 | 1.000 |
| **Static GdCl_3_ High Dose** | 183.4  ± 27.2 |  |  |  |  |  | - | **0.021** | 1.000 | 1.000 | 1.000 | 1.000 | 1.000 |
| **Dyn Nf**  **Vh Ctrl** | 251.6  ± 44.8 |  |  |  |  |  |  | - | **0.044** | **0.003** | **0.014** | **0.012** | **0.009** |
| **Dyn Nf**  **Low Dose** | 186.2  ± 48.8 |  |  |  |  |  |  |  | - | 1.000 | 1.000 | 1.000 | 1.000 |
| **Dyn Nf**  **High Dose** | 172.8  ± 27.3 |  |  |  |  |  |  |  |  | - | 1.000 | 1.000 | 1.000 |
| **Static Nf**  **Vh Ctrl** | 181.1  ± 31.8 |  |  |  |  |  |  |  |  |  | - | 1.000 | 1.000 |
| **Static Nf**  **Low Dose** | 179.9  ± 37.6 |  |  |  |  |  |  |  |  |  |  | - | 1.000 |
| **Static Nf**  **High Dose** | 178.6  ± 40.0 |  |  |  |  |  |  |  |  |  |  |  | - |

**Table S2. List of *p*-values obtained with the Tukey test for significant differences in the measurements of medial condyle height.**

|  | **Mean**  **± SD (μm)** | **Dyn GdCl_3_ Vh Ctrl** | **Dyn GdCl_3_ Low Dose** | **Dyn GdCl_3_ High Dose** | **Static GdCl_3_ Vh Ctrl** | **Static GdCl_3_ Low Dose** | **Static GdCl_3_ High Dose** | **Dyn**  **Nf**  **Vh Ctrl** | **Dyn**  **Nf**  **Low Dose** | **Dyn**  **Nf**  **High Dose** | **Static Nf**  **Vh Ctrl** | **Static Nf**  **Low Dose** | **Static Nf**  **High Dose** |
| --- | --- | --- | --- | --- | --- | --- | --- | --- | --- | --- | --- | --- | --- |
| **Mean**  **± SD (μm)** |  | 478.9  ± 94.5 | 512.9  ± 98.3 | 428.4  ± 38.2 | 447.4  ± 55.7 | 421.4  ± 50.4 | 417.7  ± 60.7 | 494.9  ± 54.0 | 486.0  ± 59.8 | 337.8  ± 64.6 | 384.1  ± 71.9 | 381.8  ± 49.1 | 386.1  ± 59.5 |
| **Dyn GdCl_3_**  **Vh Ctrl** | 478.9  ± 94.5 | - | 0.994 | 0.89 | 0.998 | 0.775 | 0.701 | 1.000 | 1.000 | **<0.001** | 0.103 | 0.085 | 0.121 |
| **Dyn GdCl_3_**  **Low Dose** | 512.9  ± 98.3 |  | - | 0.222 | 0.607 | 0.134 | 0.100 | 1.000 | 0.999 | **<0.001** | **0.004** | **0.003** | **0.005** |
| **Dyn GdCl_3_**  **High Dose** | 428.4  ± 38.2 |  |  | - | 1.000 | 1.000 | 1.000 | 0.583 | 0.807 | 0.144 | 0.953 | 0.933 | 0.966 |
| **Static GdCl_3_**  **Vh Ctrl** | 447.4  ± 55.7 |  |  |  | - | 0.999 | 0.995 | 0.925 | 0.987 | **0.027** | 0.655 | 0.602 | 0.699 |
| **Static GdCl_3_**  **Low Dose** | 421.4  ± 50.4 |  |  |  |  | - | 1.000 | 0.425 | 0.669 | 0.236 | 0.987 | 0.979 | 0.992 |
| **Static GdCl_3_ High Dose** | 417.7  ± 60.7 |  |  |  |  |  | - | 0.350 | 0.590 | 0.298 | 0.994 | 0.990 | 0.997 |
| **Dyn Nf**  **Vh Ctrl** | 494.9  ± 54.0 |  |  |  |  |  |  | - | 1.000 | **<0.001** | **0.025** | **0.019** | **0.030** |
| **Dyn Nf**  **Low Dose** | 486.0  ± 59.8 |  |  |  |  |  |  |  | - | **<0.001** | 0.074 | 0.061 | 0.087 |
| **Dyn Nf**  **High Dose** | 337.8  ± 64.6 |  |  |  |  |  |  |  |  | - | 0.936 | 0.955 | 0.916 |
| **Static Nf**  **Vh Ctrl** | 384.1  ± 71.9 |  |  |  |  |  |  |  |  |  | - | 1.000 | 1.000 |
| **Static Nf**  **Low Dose** | 381.8  ± 49.1 |  |  |  |  |  |  |  |  |  |  | - | 1.000 |
| **Static Nf**  **High Dose** | 386.1  ± 59.5 |  |  |  |  |  |  |  |  |  |  |  | - |

**Table S3. List of *p*-values obtained with the Tukey test for significant differences in the measurements of medial condyle depth.**

|  | **Mean**  **± SD (μm)** | **Dyn GdCl_3_ Vh Ctrl** | **Dyn GdCl_3_ Low Dose** | **Dyn GdCl_3_ High Dose** | **Static GdCl_3_ Vh Ctrl** | **Static GdCl_3_ Low Dose** | **Static GdCl_3_ High Dose** | **Dyn**  **Nf**  **Vh Ctrl** | **Dyn**  **Nf**  **Low Dose** | **Dyn**  **Nf**  **High Dose** | **Static Nf**  **Vh Ctrl** | **Static Nf**  **Low Dose** | **Static Nf**  **High Dose** |
| --- | --- | --- | --- | --- | --- | --- | --- | --- | --- | --- | --- | --- | --- |
| **Mean**  **± SD (μm)** |  | 458.0  ± 49.7 | 478.8  ± 72.0 | 390.6  ± 76.1 | 397.0  ± 49.0 | 392.7  ± 46.9 | 388.0  ± 39.6 | 425.5  ± 64.2 | 434.3  ± 62.3 | 320.4  ± 48.6 | 346.7  ± 69.2 | 347.7  ± 63.3 | 337.0  ± 53.8 |
| **Dyn GdCl_3_**  **Vh Ctrl** | 458.0  ± 49.7 | - | 1.000 | 0.398 | 0.555 | 0.448 | 0.340 | 0.990 | 1.000 | **<0.001** | **0.007** | **0.007** | **0.002** |
| **Dyn GdCl_3_**  **Low Dose** | 478.8  ± 72.0 |  | - | 0.080 | 0.141 | 0.097 | 0.062 | 0.743 | 0.920 | **<0.001** | **<0.001** | **<0.001** | **<0.001** |
| **Dyn GdCl_3_**  **High Dose** | 390.6  ± 76.1 |  |  | - | 1.000 | 1.000 | 1.000 | 0.982 | 0.930 | 0.336 | 0.912 | 0.924 | 0.736 |
| **Static GdCl_3_**  **Vh Ctrl** | 397.0  ± 49.0 |  |  |  | - | 1.000 | 1.000 | 0.997 | 0.977 | 0.215 | 0.808 | 0.828 | 0.581 |
| **Static GdCl_3_**  **Low Dose** | 392.7  ± 46.9 |  |  |  |  | - | 1.000 | 0.989 | 0.949 | 0.293 | 0.883 | 0.898 | 0.688 |
| **Static GdCl_3_ High Dose** | 388.0  ± 39.6 |  |  |  |  |  | - | 0.970 | 0.898 | 0.393 | 0.941 | 0.950 | 0.793 |
| **Dyn Nf**  **Vh Ctrl** | 425.5  ± 64.2 |  |  |  |  |  |  | - | 1.000 | **0.014** | 0.181 | 0.196 | 0.077 |
| **Dyn Nf**  **Low Dose** | 434.3  ± 62.3 |  |  |  |  |  |  |  | - | **0.007** | 0.108 | 0.118 | **0.044** |
| **Dyn Nf**  **High Dose** | 320.4  ± 48.6 |  |  |  |  |  |  |  |  | - | 0.998 | 0.998 | 1.000 |
| **Static Nf**  **Vh Ctrl** | 346.7  ± 69.2 |  |  |  |  |  |  |  |  |  | - | 1.000 | 1.000 |
| **Static Nf**  **Low Dose** | 347.7  ± 63.3 |  |  |  |  |  |  |  |  |  |  | - | 1.000 |
| **Static Nf**  **High Dose** | 337.0  ± 53.8 |  |  |  |  |  |  |  |  |  |  |  | - |

**Table S4. List of *p*-values obtained with the Tukey test for significant differences in the measurements of lateral condyle width.**

|  | **Mean**  **± SD (μm)** | **Dyn GdCl_3_ Vh Ctrl** | **Dyn GdCl_3_ Low Dose** | **Dyn GdCl_3_ High Dose** | **Static GdCl_3_ Vh Ctrl** | **Static GdCl_3_ Low Dose** | **Static GdCl_3_ High Dose** | **Dyn**  **Nf**  **Vh Ctrl** | **Dyn**  **Nf**  **Low Dose** | **Dyn**  **Nf**  **High Dose** | **Static Nf**  **Vh Ctrl** | **Static Nf**  **Low Dose** | **Static Nf**  **High Dose** |
| --- | --- | --- | --- | --- | --- | --- | --- | --- | --- | --- | --- | --- | --- |
| **Mean**  **± SD (μm)** |  | 432.2  ± 42.1 | 438.0  ± 67.0 | 461.0  ± 70.7 | 401.2  ± 42.2 | 397.1  ± 42.6 | 406.8  ± 38.4 | 487.6  ± 70.6 | 475.8  ± 76.6 | 409.3  ± 31.1 | 408.3  ± 64.8 | 400.5  ± 46.7 | 398.1  ± 35.6 |
| **Dyn GdCl_3_**  **Vh Ctrl** | 432.2  ± 42.1 | - | 1.000 | 0.993 | 0.987 | 0.966 | 0.998 | 0.578 | 0.884 | 0.999 | 0.999 | 0.985 | 0.973 |
| **Dyn GdCl_3_**  **Low Dose** | 438.1  ± 67.0 |  | - | 0.999 | 0.953 | 0.906 | 0.986 | 0.732 | 0.953 | 0.993 | 0.991 | 0.947 | 0.920 |
| **Dyn GdCl_3_**  **High Dose** | 461.0  ± 70.7 |  |  | - | 0.458 | 0.354 | 0.611 | 0.996 | 1.000 | 0.679 | 0.652 | 0.442 | 0.380 |
| **Static GdCl_3_**  **Vh Ctrl** | 401.2  ± 42.2 |  |  |  | - | 1.000 | 1.000 | **0.047** | 0.185 | 1.000 | 1.000 | 1.000 | 1.000 |
| **Static GdCl_3_**  **Low Dose** | 397.1  ± 42.6 |  |  |  |  | - | 1.000 | **0.029** | 0.130 | 1.000 | 1.000 | 1.000 | 1.000 |
| **Static GdCl_3_ High Dose** | 406.8  ± 38.4 |  |  |  |  |  | - | 0.085 | 0.286 | 1.000 | 1.000 | 1.000 | 1.000 |
| **Dyn Nf**  **Vh Ctrl** | 487.6  ± 70.6 |  |  |  |  |  |  | - | 1.000 | 0.108 | 0.098 | **0.044** | **0.033** |
| **Dyn Nf**  **Low Dose** | 475.8  ± 76.6 |  |  |  |  |  |  |  | - | 0.341 | 0.318 | 0.176 | 0.143 |
| **Dyn Nf**  **High Dose** | 409.3  ± 31.1 |  |  |  |  |  |  |  |  | - | 1.000 | 1.000 | 1.000 |
| **Static Nf**  **Vh Ctrl** | 408.3  ± 64.8 |  |  |  |  |  |  |  |  |  | - | 1.000 | 1.000 |
| **Static Nf**  **Low Dose** | 400.5  ± 46.7 |  |  |  |  |  |  |  |  |  |  | - | 1.000 |
| **Static Nf**  **High Dose** | 398.1  ± 35.6 |  |  |  |  |  |  |  |  |  |  |  | - |

**Table S5. List of *p*-values obtained with the Tukey test for significant differences in the measurements of lateral condyle height.**

|  | **Mean**  **± SD (μm)** | **Dyn GdCl_3_ Vh Ctrl** | **Dyn GdCl_3_ Low Dose** | **Dyn GdCl_3_ High Dose** | **Static GdCl_3_ Vh Ctrl** | **Static GdCl_3_ Low Dose** | **Static GdCl_3_ High Dose** | **Dyn**  **Nf**  **Vh Ctrl** | **Dyn**  **Nf**  **Low Dose** | **Dyn**  **Nf**  **High Dose** | **Static Nf**  **Vh Ctrl** | **Static Nf**  **Low Dose** | **Static Nf**  **High Dose** |
| --- | --- | --- | --- | --- | --- | --- | --- | --- | --- | --- | --- | --- | --- |
| **Mean**  **± SD (μm)** |  | 823.0  ± 100.2 | 748.8  ± 102.3 | 755.3  ± 129.7 | 737.3  ± 50.2 | 723.9  ± 42.9 | 730.5  ± 32.5 | 840.4  ± 64.9 | 845.2  ± 95.0 | 666.0  ± 116.2 | 706.7  ± 61.3 | 707.4  ± 47.0 | 698.6  ± 72.2 |
| **Dyn GdCl_3_**  **Vh Ctrl** | 823.0  ± 100.2 | - | 0.741 | 0.836 | 0.538 | 0.311 | 0.417 | 1.000 | 1.000 | **0.005** | 0.120 | 0.125 | 0.070 |
| **Dyn GdCl_3_**  **Low Dose** | 748.8  ± 102.3 |  | - | 1.000 | 1.000 | 1.000 | 1.000 | 0.434 | 0.401 | 0.592 | 0.994 | 0.995 | 0.977 |
| **Dyn GdCl_3_**  **High Dose** | 755.2  ± 129.7 |  |  | - | 1.000 | 1.000 | 1.000 | 0.549 | 0.511 | 0.475 | 0.982 | 0.984 | 0.945 |
| **Static GdCl_3_**  **Vh Ctrl** | 737.3  ± 50.2 |  |  |  | - | 1.000 | 1.000 | 0.257 | 0.237 | 0.797 | 1.000 | 1.000 | 0.997 |
| **Static GdCl_3_**  **Low Dose** | 723.9  ± 42.9 |  |  |  |  | - | 1.000 | 0.119 | 0.110 | 0.937 | 1.000 | 1.000 | 1.000 |
| **Static GdCl_3_ High Dose** | 730.5  ± 32.5 |  |  |  |  |  | - | 0.177 | 0.163 | 0.877 | 1.000 | 1.000 | 1.000 |
| **Dyn Nf**  **Vh Ctrl** | 840.4  ± 64.9 |  |  |  |  |  |  | - | 1.000 | **0.001** | **0.036** | **0.038** | **0.019** |
| **Dyn Nf**  **Low Dose** | 845.2  ± 95.0 |  |  |  |  |  |  |  | - | **0.001** | **0.034** | **0.036** | **0.018** |
| **Dyn Nf**  **High Dose** | 666.0  ± 116.2 |  |  |  |  |  |  |  |  | - | 0.996 | 0.995 | 0.999 |
| **Static Nf**  **Vh Ctrl** | 706.7  ± 61.3 |  |  |  |  |  |  |  |  |  | - | 1.000 | 1.000 |
| **Static Nf**  **Low Dose** | 707.4  ± 47.0 |  |  |  |  |  |  |  |  |  |  | - | 1.000 |
| **Static Nf**  **High Dose** | 698.6  ± 72.2 |  |  |  |  |  |  |  |  |  |  |  | - |

**Table S6. List of *p*-values obtained with the Tukey test for significant differences in the measurements of lateral condyle depth.**

|  | **Mean**  **± SD (μm)** | **Dyn GdCl_3_ Vh Ctrl** | **Dyn GdCl_3_ Low Dose** | **Dyn GdCl_3_ High Dose** | **Static GdCl_3_ Vh Ctrl** | **Static GdCl_3_ Low Dose** | **Static GdCl_3_ High Dose** | **Dyn**  **Nf**  **Vh Ctrl** | **Dyn**  **Nf**  **Low Dose** | **Dyn**  **Nf**  **High Dose** | **Static Nf**  **Vh Ctrl** | **Static Nf**  **Low Dose** | **Static Nf**  **High Dose** |
| --- | --- | --- | --- | --- | --- | --- | --- | --- | --- | --- | --- | --- | --- |
| **Mean**  **± SD (μm)** |  | 653.7  ± 67.5 | 633.7  ± 91.4 | 535.8  ± 82.1 | 549.5  ± 51.3 | 545.4  ± 50.2 | 547.4  ± 48.6 | 657.2  ± 69.5 | 633.4  ± 96.1 | 543.4  ± 54.0 | 533.7  ± 64.9 | 530.6  ± 42.0 | 527.8  ± 61.1 |
| **Dyn GdCl_3_**  **Vh Ctrl** | 653.7  ± 67.5 | - | 1.000 | **0.015** | 0.055 | **0.038** | **0.045** | 1.000 | 1.000 | **0.031** | **0.012** | **0.009** | **0.007** |
| **Dyn GdCl_3_**  **Low Dose** | 633.7  ± 91.4 |  | - | 0.094 | 0.252 | 0.192 | 0.220 | 1.000 | 1.000 | 0.167 | 0.079 | 0.060 | **0.047** |
| **Dyn GdCl_3_**  **High Dose** | 535.8  ± 82.1 |  |  | - | 1.000 | 1.000 | 1.000 | **0.010** | 0.121 | 1.000 | 1.000 | 1.000 | 1.000 |
| **Static GdCl_3_**  **Vh Ctrl** | 549.5  ± 51.3 |  |  |  | - | 1.000 | 1.000 | **0.040** | 0.299 | 1.000 | 1.000 | 1.000 | 1.000 |
| **Static GdCl_3_**  **Low Dose** | 545.4  ± 50.2 |  |  |  |  | - | 1.000 | **0.027** | 0.234 | 1.000 | 1.000 | 1.000 | 1.000 |
| **Static GdCl_3_ High Dose** | 547.4  ± 48.6 |  |  |  |  |  | - | **0.033** | 0.264 | 1.000 | 1.000 | 1.000 | 1.000 |
| **Dyn Nf**  **Vh Ctrl** | 657.2  ± 69.5 |  |  |  |  |  |  | - | 1.000 | **0.022** | **0.008** | **0.006** | **0.004** |
| **Dyn Nf**  **Low Dose** | 633.4  ± 96.1 |  |  |  |  |  |  |  | - | 0.206 | 0.103 | 0.080 | 0.064 |
| **Dyn Nf**  **High Dose** | 543.4  ± 54.0 |  |  |  |  |  |  |  |  | - | 1.000 | 1.000 | 1.000 |
| **Static Nf**  **Vh Ctrl** | 533.7  ± 64.9 |  |  |  |  |  |  |  |  |  | - | 1.000 | 1.000 |
| **Static Nf**  **Low Dose** | 530.6  ± 42.0 |  |  |  |  |  |  |  |  |  |  | - | 1.000 |
| **Static Nf**  **High Dose** | 527.8  ± 61.1 |  |  |  |  |  |  |  |  |  |  |  | - |

**Table S7. List of *p*-values obtained with the Kruskal-Wallis non-parametric test for significant differences in the measurements of tibia width.**

|  | **Mean**  **± SD (μm)** | **Dyn GdCl_3_ Vh Ctrl** | **Dyn GdCl_3_ Low Dose** | **Dyn GdCl_3_ High Dose** | **Static GdCl_3_ Vh Ctrl** | **Static GdCl_3_ Low Dose** | **Static GdCl_3_ High Dose** | **Dyn**  **Nf**  **Vh Ctrl** | **Dyn**  **Nf**  **Low Dose** | **Dyn**  **Nf**  **High Dose** | **Static Nf**  **Vh Ctrl** | **Static Nf**  **Low Dose** | **Static Nf**  **High Dose** |
| --- | --- | --- | --- | --- | --- | --- | --- | --- | --- | --- | --- | --- | --- |
| **Mean**  **± SD (μm)** |  | 656.6  ± 143.7 | 630.1  ± 62.6 | 652.6  ± 149.5 | 698.6  ± 52.7 | 682.3  ± 60.2 | 685.6  ± 53.0 | 723.2  ± 83.3 | 720.6  ± 70.1 | 527.6  ± 114.1 | 616.5  ± 71.6 | 618.5  ± 76.4 | 620.3  ± 73.1 |
| **Dyn GdCl_3_**  **Vh Ctrl** | 656.6  ± 143.7 | - | 1.000 | 1.000 | 1.000 | 1.000 | 1.000 | 1.000 | 1.000 | 1.000 | 1.000 | 1.000 | 1.000 |
| **Dyn GdCl_3_**  **Low Dose** | 630.1  ± 62.6 |  | - | 1.000 | 1.000 | 1.000 | 1.000 | 1.000 | 1.000 | 1.000 | 1.000 | 1.000 | 1.000 |
| **Dyn GdCl_3_**  **High Dose** | 652.6  ± 149.5 |  |  | - | 1.000 | 1.000 | 1.000 | 1.000 | 1.000 | 0.530 | 1.000 | 1.000 | 1.000 |
| **Static GdCl_3_**  **Vh Ctrl** | 698.6  ± 52.7 |  |  |  | - | 1.000 | 1.000 | 1.000 | 1.000 | 0.056 | 1.000 | 1.000 | 1.000 |
| **Static GdCl_3_**  **Low Dose** | 682.3  ± 60.2 |  |  |  |  | - | 1.000 | 1.000 | 1.000 | 0.242 | 1.000 | 1.000 | 1.000 |
| **Static GdCl_3_ High Dose** | 685.6  ± 53.0 |  |  |  |  |  | - | 1.000 | 1.000 | 0.180 | 1.000 | 1.000 | 1.000 |
| **Dyn Nf**  **Vh Ctrl** | 723.2  ± 83.3 |  |  |  |  |  |  | - | 1.000 | **0.013** | 0.867 | 1.000 | 1.000 |
| **Dyn Nf**  **Low Dose** | 720.6  ± 70.1 |  |  |  |  |  |  |  | - | **0.010** | 0.651 | 0.771 | 0.856 |
| **Dyn Nf**  **High Dose** | 527.6  ± 114.1 |  |  |  |  |  |  |  |  | - | 1.000 | 1.000 | 1.000 |
| **Static Nf**  **Vh Ctrl** | 616.5  ± 71.6 |  |  |  |  |  |  |  |  |  | - | 1.000 | 1.000 |
| **Static Nf**  **Low Dose** | 618.5  ± 76.4 |  |  |  |  |  |  |  |  |  |  | - | 1.000 |
| **Static Nf**  **High Dose** | 620.3  ± 73.1 |  |  |  |  |  |  |  |  |  |  |  | - |

**Table S8. List of *p*-values obtained with the Tukey test for significant differences in the measurements of tibia depth.**

|  | **Mean**  **± SD (μm)** | **Dyn GdCl_3_ Vh Ctrl** | **Dyn GdCl_3_ Low Dose** | **Dyn GdCl_3_ High Dose** | **Static GdCl_3_ Vh Ctrl** | **Static GdCl_3_ Low Dose** | **Static GdCl_3_ High Dose** | **Dyn**  **Nf**  **Vh Ctrl** | **Dyn**  **Nf**  **Low Dose** | **Dyn**  **Nf**  **High Dose** | **Static Nf**  **Vh Ctrl** | **Static Nf**  **Low Dose** | **Static Nf**  **High Dose** |
| --- | --- | --- | --- | --- | --- | --- | --- | --- | --- | --- | --- | --- | --- |
| **Mean**  **± SD (μm)** |  | 1097.1  ± 104.1 | 1089.1  ± 40.4 | 852.3  ± 93.6 | 879.7  ± 61.7 | 881.8  ± 54.0 | 894.6  ± 48.4 | 1100.2  ± 90.7 | 1045.4  ± 114.0 | 830.0  ± 75.3 | 877.5  ± 74.2 | 865.2  ± 70.5 | 857.4  ± 55.2 |
| **Dyn GdCl_3_**  **Vh Ctrl** | 1097.1  ± 104.1 | - | 1.000 | **<0.001** | **<0.001** | **<0.001** | **<0.001** | 1.000 | 0.962 | **<0.001** | **<0.001** | **<0.001** | **<0.001** |
| **Dyn GdCl_3_**  **Low Dose** | 1089.1  ± 40.4 |  | - | **<0.001** | **<0.001** | **<0.001** | **<0.001** | 1.000 | 0.990 | **<0.001** | **<0.001** | **<0.001** | **<0.001** |
| **Dyn GdCl_3_**  **High Dose** | 852.3  ± 93.6 |  |  | - | 1.000 | 1.000 | 0.990 | **<0.001** | **<0.001** | 1.000 | 1.000 | 1.000 | 1.000 |
| **Static GdCl_3_**  **Vh Ctrl** | 879.7  ± 61.7 |  |  |  | - | 1.000 | 1.000 | **<0.001** | **0.001** | 0.964 | 1.000 | 1.000 | 1.000 |
| **Static GdCl_3_**  **Low Dose** | 881.8  ± 54.0 |  |  |  |  | - | 1.000 | **<0.001** | **0.002** | 0.952 | 1.000 | 1.000 | 1.000 |
| **Static GdCl_3_ High Dose** | 894.6  ± 48.4 |  |  |  |  |  | - | **<0.001** | **0.005** | 0.815 | 1.000 | 1.000 | 0.997 |
| **Dyn Nf**  **Vh Ctrl** | 1100.2  ± 90.7 |  |  |  |  |  |  | - | 0.943 | **<0.001** | **<0.001** | **<0.001** | **<0.001** |
| **Dyn Nf**  **Low Dose** | 1045.4  ± 114.0 |  |  |  |  |  |  |  | - | **<0.001** | **0.001** | **<0.001** | **<0.001** |
| **Dyn Nf**  **High Dose** | 830.0  ± 75.3 |  |  |  |  |  |  |  |  | - | 0.974 | 0.998 | 1.000 |
| **Static Nf**  **Vh Ctrl** | 877.5  ± 74.2 |  |  |  |  |  |  |  |  |  | - | 1.000 | 1.000 |
| **Static Nf**  **Low Dose** | 865.2  ± 70.5 |  |  |  |  |  |  |  |  |  |  | - | 1.000 |
| **Static Nf**  **High Dose** | 857.4  ± 55.2 |  |  |  |  |  |  |  |  |  |  |  | - |

**Table S9. List of *p*-values obtained with the Kruskal-Wallis non-parametric test for significant differences in the measurements of fibula depth.**

|  | **Mean**  **± SD (μm)** | **Dyn GdCl_3_ Vh Ctrl** | **Dyn GdCl_3_ Low Dose** | **Dyn GdCl_3_ High Dose** | **Static GdCl_3_ Vh Ctrl** | **Static GdCl_3_ Low Dose** | **Static GdCl_3_ High Dose** | **Dyn**  **Nf**  **Vh Ctrl** | **Dyn**  **Nf**  **Low Dose** | **Dyn**  **Nf**  **High Dose** | **Static Nf**  **Vh Ctrl** | **Static Nf**  **Low Dose** | **Static Nf**  **High Dose** |
| --- | --- | --- | --- | --- | --- | --- | --- | --- | --- | --- | --- | --- | --- |
| **Mean**  **± SD (μm)** |  | 510.5  ± 63.9 | 476.1  ± 41.1 | 503.8  ± 80.3 | 449.2  ± 41.7 | 453.7  ± 42.2 | 454.4  ± 37.6 | 540.9  ± 58.2 | 557.1  ± 35.8 | 479.4  ± 79.7 | 470.1  ± 33.8 | 466.0  ± 57.3 | 458.8  ± 32.9 |
| **Dyn GdCl_3_**  **Vh Ctrl** | 510.5  ± 63.9 | - | 1.000 | 1.000 | 1.000 | 1.000 | 1.000 | 1.000 | 1.000 | 1.000 | 1.000 | 1.000 | 1.000 |
| **Dyn GdCl_3_**  **Low Dose** | 476.1  ± 41.1 |  | - | 1.000 | 1.000 | 1.000 | 1.000 | 1.000 | 0.348 | 1.000 | 1.000 | 1.000 | 1.000 |
| **Dyn GdCl_3_**  **High Dose** | 503.8  ± 80.3 |  |  | - | 1.000 | 1.000 | 1.000 | 1.000 | 1.000 | 1.000 | 1.000 | 1.000 | 1.000 |
| **Static GdCl_3_**  **Vh Ctrl** | 449.2  ± 41.7 |  |  |  | - | 1.000 | 1.000 | 0.084 | **0.012** | 1.000 | 1.000 | 1.000 | 1.000 |
| **Static GdCl_3_**  **Low Dose** | 453.7  ± 42.2 |  |  |  |  | - | 1.000 | 0.123 | **0.018** | 1.000 | 1.000 | 1.000 | 1.000 |
| **Static GdCl_3_ High Dose** | 454.4  ± 37.6 |  |  |  |  |  | - | 0.128 | **0.019** | 1.000 | 1.000 | 1.000 | 1.000 |
| **Dyn Nf**  **Vh Ctrl** | 540.9  ± 58.2 |  |  |  |  |  |  | - | 1.000 | 1.000 | 0.805 | 1.000 | 0.197 |
| **Dyn Nf**  **Low Dose** | 557.1  ± 35.8 |  |  |  |  |  |  |  | - | 1.000 | 0.148 | 0.252 | **0.030** |
| **Dyn Nf**  **High Dose** | 479.4  ± 79.7 |  |  |  |  |  |  |  |  | - | 1.000 | 1.000 | 1.000 |
| **Static Nf**  **Vh Ctrl** | 470.1  ± 33.8 |  |  |  |  |  |  |  |  |  | - | 1.000 | 1.000 |
| **Static Nf**  **Low Dose** | 466.0  ± 57.3 |  |  |  |  |  |  |  |  |  |  | - | 1.000 |
| **Static Nf**  **High Dose** | 458.8  ± 32.9 |  |  |  |  |  |  |  |  |  |  |  | - |

**Table S10. List of *p*-values obtained with the Tukey test for significant differences in the measurements of intercondylar fossa width.**

|  | **Mean**  **± SD (μm)** | **Dyn GdCl_3_ Vh Ctrl** | **Dyn GdCl_3_ Low Dose** | **Dyn GdCl_3_ High Dose** | **Static GdCl_3_ Vh Ctrl** | **Static GdCl_3_ Low Dose** | **Static GdCl_3_ High Dose** | **Dyn**  **Nf**  **Vh Ctrl** | **Dyn**  **Nf**  **Low Dose** | **Dyn**  **Nf**  **High Dose** | **Static Nf**  **Vh Ctrl** | **Static Nf**  **Low Dose** | **Static Nf**  **High Dose** |
| --- | --- | --- | --- | --- | --- | --- | --- | --- | --- | --- | --- | --- | --- |
| **Mean**  **± SD (μm)** |  | 192.2  ± 31.3 | 177.6  ± 41.5 | 163.9  ± 54.0 | 173.0  ± 44.3 | 163.5  ± 35.3 | 159.9  ± 41.6 | 221.3  ± 27.5 | 214.2  ± 65.5 | 147.0  ± 33.9 | 161.4  ± 30.3 | 157.2  ± 36.5 | 155.1  ± 31.3 |
| **Dyn GdCl_3_**  **Vh Ctrl** | 192.2  ± 31.3 | - | 1.000 | 0.941 | 0.997 | 0.935 | 0.868 | 0.929 | 0.993 | 0.438 | 0.900 | 0.794 | 0.727 |
| **Dyn GdCl_3_**  **Low Dose** | 177.6  ± 41.5 |  | - | 1.000 | 1.000 | 1.000 | 0.999 | 0.488 | 0.778 | 0.905 | 0.999 | 0.995 | 0.989 |
| **Dyn GdCl_3_**  **High Dose** | 163.9  ± 54.0 |  |  | - | 1.000 | 1.000 | 1.000 | 0.121 | 0.317 | 0.999 | 1.000 | 1.000 | 1.000 |
| **Static GdCl_3_**  **Vh Ctrl** | 173.0  ± 44.3 |  |  |  | - | 1.000 | 1.000 | 0.333 | 0.627 | 0.968 | 1.000 | 1.000 | 0.999 |
| **Static GdCl_3_**  **Low Dose** | 163.5  ± 35.3 |  |  |  |  | - | 1.000 | 0.115 | 0.305 | 0.999 | 1.000 | 1.000 | 1.000 |
| **Static GdCl_3_ High Dose** | 159.9  ± 41.6 |  |  |  |  |  | - | 0.072 | 0.214 | 1.000 | 1.000 | 1.000 | 1.000 |
| **Dyn Nf**  **Vh Ctrl** | 221.3  ± 27.5 |  |  |  |  |  |  | - | 1.000 | **0.010** | 0.088 | **0.048** | **0.035** |
| **Dyn Nf**  **Low Dose** | 214.2  ± 65.5 |  |  |  |  |  |  |  | - | **0.042** | 0.250 | 0.158 | 0.122 |
| **Dyn Nf**  **High Dose** | 147.0  ± 33.9 |  |  |  |  |  |  |  |  | - | 1.000 | 1.000 | 1.000 |
| **Static Nf**  **Vh Ctrl** | 161.4  ± 30.3 |  |  |  |  |  |  |  |  |  | - | 1.000 | 1.000 |
| **Static Nf**  **Low Dose** | 157.2  ± 36.5 |  |  |  |  |  |  |  |  |  |  | - | 1.000 |
| **Static Nf**  **High Dose** | 155.1  ± 31.3 |  |  |  |  |  |  |  |  |  |  |  | - |
